# Sodium *versus* Potassium Salts

**Published:** 1890-05-03

**Authors:** 


### THERAPEUTICS.

### SODIUM *VERSUS* POTASSIUM SALTS.

Dr. Laborde protests against the assumption that salts, having allied basic or halogen factors, may be indiscriminately substituted one for the other. Not only are the actions of the bromides and iodides almost, if not quite, as diverse as those of the chlorides are from either, but he maintains that the therapeutic values of the chlorates, and what is of more importance, of the iodides of sodium are far less than those of potassium. He has seen benefit follow almost immediately on the substitution of iodide of potassium, when the sodium salt had been given for a long time without any result. A comparison of the relative actions of the iodides and bromides of potassium, sodium, and ammonium is still to be desired.

